# Supplementary material for: Optimizing the internal phase reference to shape the output of a multimode optical fiber
Source: PLoS One. 2023 Sep 8;18(9):e0290300. doi: 10.1371/journal.pone.0290300 (PMC10490902; doi:10.1371/journal.pone.0290300)
Supplement: S1 File — (DOCX) [file pone.0290300.s001.docx]

Supporting figures


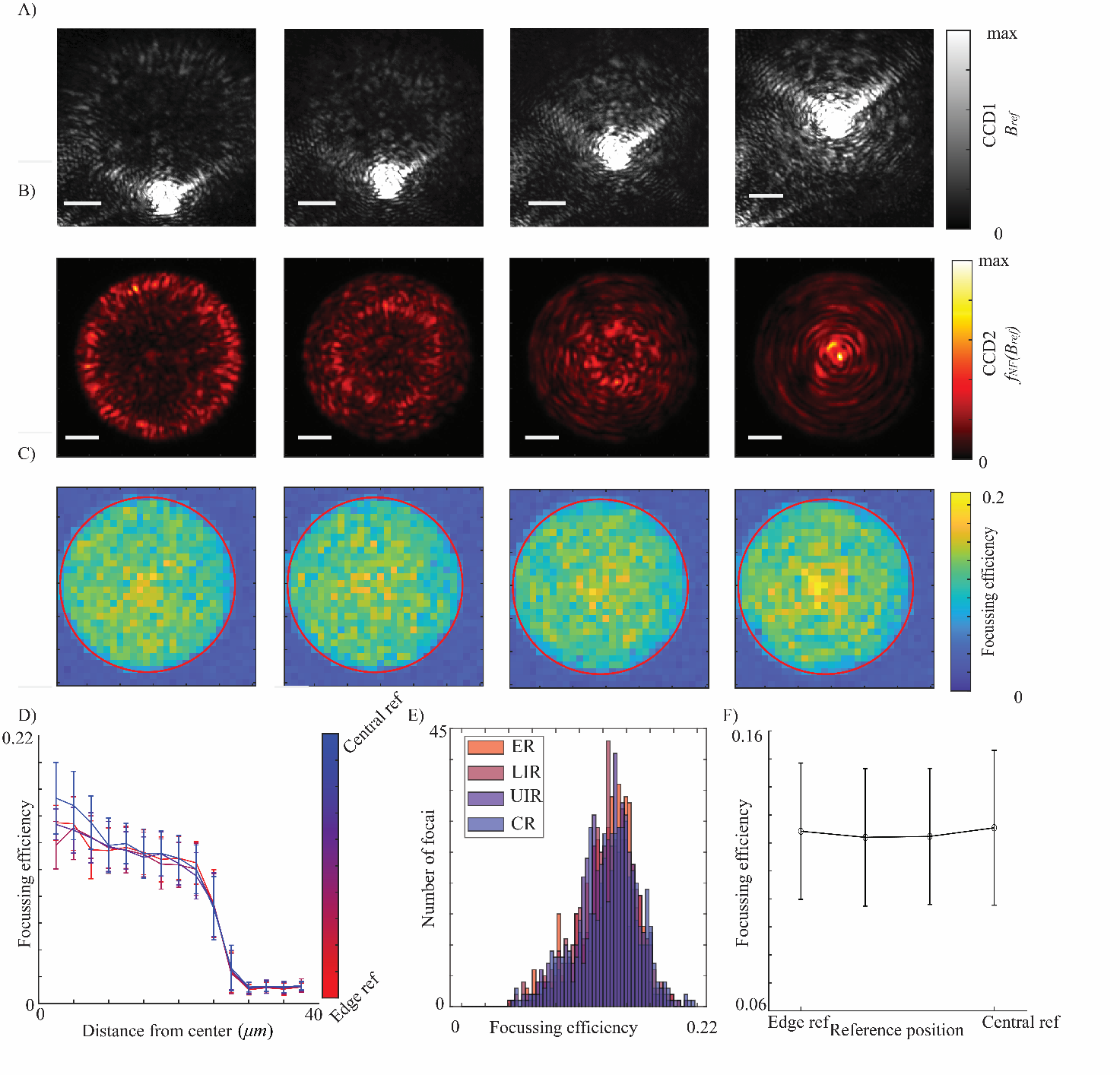


***Figure S1*** *– Analysis of calibrations based on internal reference beams through a 60 cm long fiber. a) Four internal reference beams at the input facet* $B_{ref}^{i}$*(scale bar 10*$\mu$*m). B) The transmitted internal reference beams* $f_{FP}(B_{ref}^{i} )$ *imaged on CCD2, the fiber core is shown as a red circle. C) Focussing efficiency across the fiber core for each calibration D) Dependence of focussing efficiency on distance from center of the core. E) Histograms of focussing efficiency (for foci within the fiber core). F) The fall in average focusing efficiency between edge and centre reference based calibrations.*


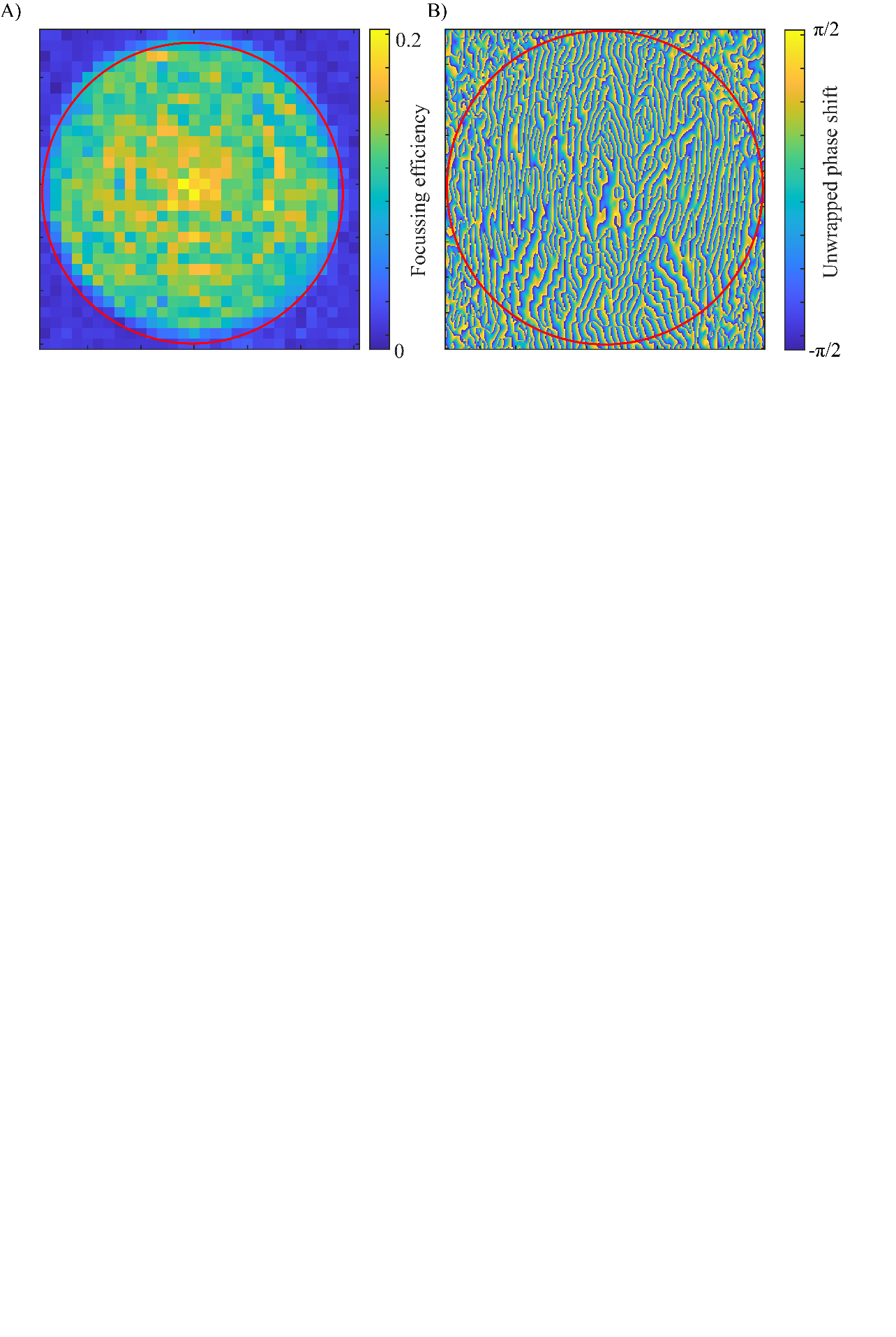


***Figure S2*** *– A) The resultant focussing efficiency map from an internal reference (edge) based calibration. B) The phase profile of the internal reference measured by a phase unwrapping of the interferogram between the internal and external reference beams.*


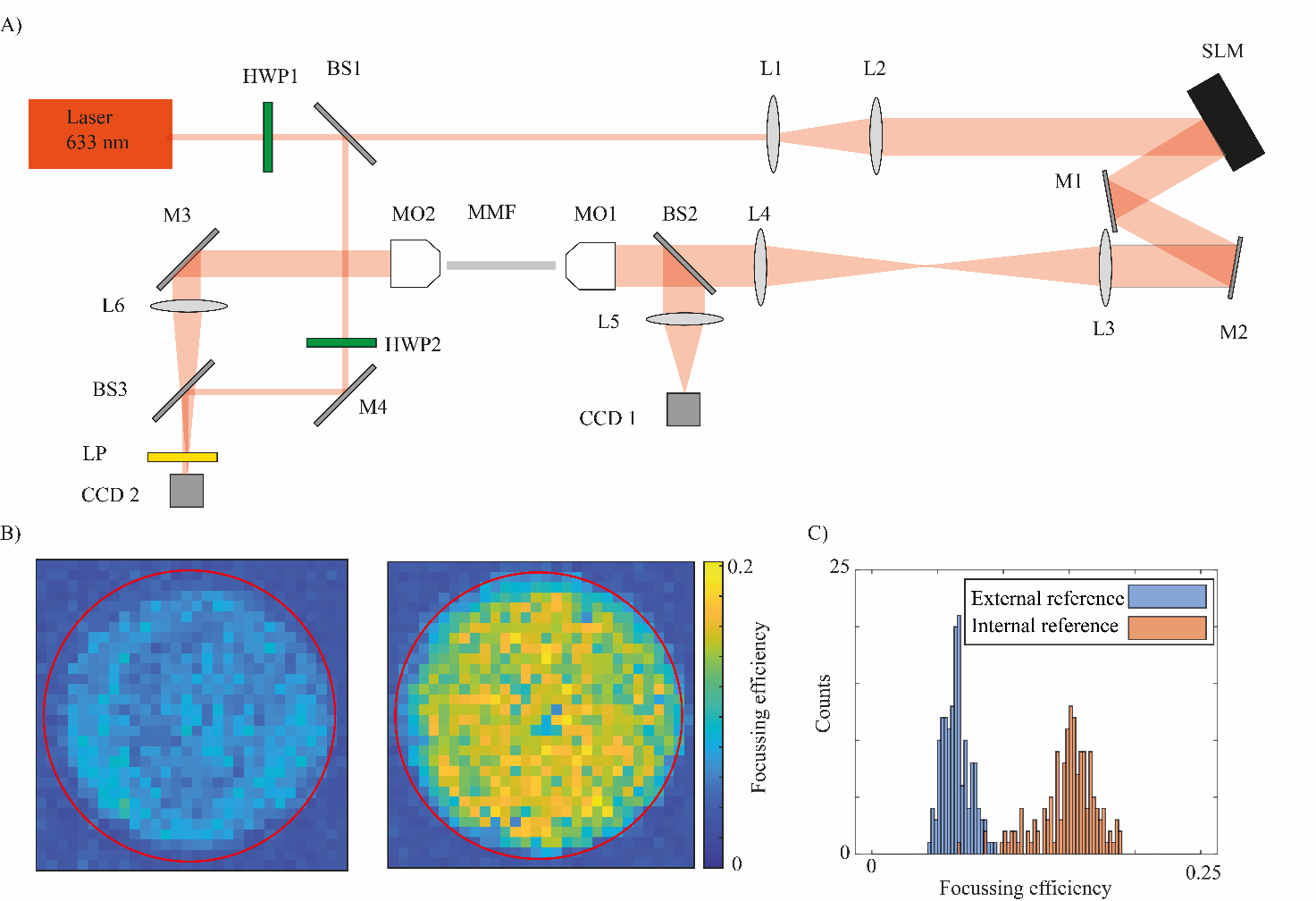


***Figure S3****– A comparison between internal reference and external reference based calibrations. A) Optical system for shaping light at the tip of a MMF using an internal reference. HWP- half wave plater, L- lens, SLM- spatial light modulator, M- mirror, BS- beam splitter, MO - microscope objective, MMF – multimode fiber, LP- Linear polarisers, CCD- charged coupling device. B) Focussing efficiency maps for external (left) and internal reference based measurements (right) C) Histogram of B).*
