# Supplementary material for: Optimizing the internal phase reference to shape the output of a multimode optical fiber
Source: PLoS One. 2023 Sep 8;18(9):e0290300. doi: 10.1371/journal.pone.0290300 (PMC10490902; doi:10.1371/journal.pone.0290300)
Supplement: Attachment — Submitted filename: my review.docx [file pone.0290300.s002.docx]

In the manuscript tilted “Determining the optimal reference beam to measure the transmission matrix of a multimode optical fiber”, the authors present the merits of using internal reference method to improve focusing through a multimode fiber. The internal reference method is a great idea in my opinion, but the work presented by the author needs to ‘dig a bit deeper’.

The best way to generate a single focus using wavefront shaping method is the step sequential method introduced at the dawn of the technique. The transmission matrix method is the most accurate but it requires determining both amplitude and phase of the transmitted signal. The most accurate method to obtained the TM uses some sort of external/stable reference beam (holographic method) or uses phase retrieval techniques (a bit more computationally intensive). All the above methods can generate/transfer images through multimode fibers.

Here the authors only demonstrate their work for a single focus. I believe Collard *et al.* will have to put their work more in context to justify this conclusion “*Overall, our study demonstrates the utility of internal reference-based systems, which is inherently reconfigurable, we envisage this will be crucial in allowing the technology to reach its full potential for hyper-spectral imaging at the tip of a hair thin optical fiber.”*

See below for further questions and concerns:

1. The authors can do a little bit more of digging in the literature. There is quite a comprehensive body of work for wavefront shaping in multimode fiber both with external reference, internal reference, and reference-less (phase retrieval). How is this method compare with the other well established endoscopic imaging method?
2. The authors should revised their references. For example Reference [24] is not a reference less method. They have an external reference going through a second fiber. I do not believe they claim AI. The references for [25] and [26] aren’t accurate either. I believe the numbers have been shifted down. Please revise.
3. The length of the fiber (4 cm) used in this experiment is of concern. As far as I know, a lot of the single fiber endoscopic method in the literature have much longer fibers, some with much larger core (more modes). How will this method presented here, work when the fiber is 10 or 20 times longer? This isn’t obvious to me; in a longer fiber the light are even more scrambled. Further, bends, temperature, vibrations, etc. will also affect both the signal and reference. How is this accounted for in this study?
4. Further, the most accurate holographic techniques use a spatially filtered external reference beam: the phase and intensity of the beam well controlled and known. The idea of an internal reference is indeed fascinating, but the internal reference will also suffer the same fate as the signal beam. Can the author discuss how is the amplitude and phase of the internal reference determined? In equation (3) in the manuscript, the intensity of the reference beam is measured accurately, but how is the phase determined?
5. How is the work presented in this manuscript improve from what has been done in reference [26]? What is novel? Different?
6. There is a discussion on a single focus. Wavefront shaping have significantly moved on to multiple foci and image transfer. How will the changes in a multiple internal reference affect a multiple foci problem? An image?
