# Supplementary material for: Optimizing the internal phase reference to shape the output of a multimode optical fiber
Source: PLoS One. 2023 Sep 8;18(9):e0290300. doi: 10.1371/journal.pone.0290300 (PMC10490902; doi:10.1371/journal.pone.0290300)
Supplement: Attachment — Submitted filename: ResponseLetterFINAL.docx [file pone.0290300.s003.docx]

REVIEWER 1

**COMMENT:**

*In this paper, a reference beam optimization scheme for measuring transmission matrix of multimode fiber based on the internal reference beam is proposed through experiments and analysis. By changing the coupling position and number of the internal reference beam and analyzing the speckle distribution and Fourier transform spectral intensity of the output facet of optical fiber, the author summarizes the implementation scheme of the inner reference beam. This manuscript is written logically and clearly shown the purpose. It may be accepted for publishing in PONE after a minor revision, but there are still several problems.*

**REPLY:**

We thank the reviewer for the positive opinion on the work and the constructive comments, which have helped us in improving the overall readability and quality of the manuscript. Our detailed answers are reported below.

**QUESTION R1.1:** *The author said “The numerical aperture of MO1 was significantly higher than that of the MMF. This was done so that the input modes of the fiber could be fully sampled”. Please explain that in detail.*

**REPLY R1.1:**

We thank the reviewer for letting us discuss this point. The number of modes supported by the fiber is given by $M=\frac{V^{2}}{2}$ where $V=\frac{2\pi r}{\lambda}NA=\frac{2\times\pi\times25}{0.633}\times0.22$=54.59. Thus, $M\approx1490$ (or 745 per polarisation), and following (Cizmar. T., Dholakia. K., Optics Express 2011) the number of points sampled on the input facet (i.e. pairs (*j_x_, j_y_*)) should roughly correspond with this number as pairs (*j_x_, j_y_*) correspond to a basis for guided modes within the fiber. Therefore, the minimum lateral resolution at the input should be $\frac{2r}{\sqrt{M_{p}}}=\frac{50\mu m}{27.3}=1.83 um$. Meanwhile, Abbe’s diffraction formula for lateral (XY) resolution calculated with MO1 is $d = \frac{\lambda}{2NA}=\frac{.633}{2*0.65}=0.48 um$. The above calculation has been added to the text:

*“The numerical aperture of MO1 was significantly higher than that of the MMF. This was done so as the number of pairs (j_x_, j_y_) should roughly match the number of modes carried by the fiber  [2]. The number of modes supported by the fiber is given by* $M=\frac{V^{2}}{2}$ *where* $V=\frac{2\pi r}{\lambda}NA=\frac{2\times\pi\times25}{0.633}\times0.22$*=54.59. Thus,* $M\approx1490$ *(or 745 per polarisation). Therefore, the minimum lateral resolution at the input should be* $\frac{2r}{\sqrt{M_{p}}}=\frac{50\mu m}{27.3}=1.83 um$*. Meanwhile, Abbe’s diffraction formula for lateral (XY) resolution calculated with MO1 is* $d = \frac{\lambda}{2NA}=\frac{.633}{2*0.65}=0.48 um$*. The transmission efficiency from MO1 to the MMF is approximately 30%”.*

**QUESTION R1.2:** *It seems that the choice of reference beam can be determined from the analysis of the output face of the fiber. Why do we need to do Fourier analysis?*

**REPLY R1.2:**

Through the article we work both on the surface of the fiber *(x_out_, y_out_*) plane and in the fourier plane (*u_out_, v_out_*), since both are relevant for endoscopic applications *(T. Čižmár and K. Dholakia, "Exploiting multimode waveguides for pure fibre-based imaging," Nat. Commun. 3, 1027 (2012).)* and *(I. T. Leite, S. Turtaev, D. E. Boonzajer Flaes, and T. Čižmár, "Observing distant objects with a multimode fiber-based holographic endoscope," APL Photonics 6, 36112 (2021).):* *(x_out_, y_out_*) to image in the vicinity, while (*u_out_, v_out_*) to sense at longer distances from the facet. The final sentence of the introduction has been modified to read:

*“In this work we aim to provide a detailed assessment of both surface and Fourier plane calibrations (where the light is focussed on the distal facet or gets collimated in a beam of low divergence emerging from the distal facet, respectively) performed with single and serialised internal reference beams, striving at optimizing the relative focussing efficiency of the system .”*

**QUESTION R1.3:** *Obviously, in order to record the speckle field completely, the speckle field of the reference beam must be uniform enough. So, are there other factors other than the appropriate focusing position described in the article? Such as numerical aperture of MO, quality of the reference beam, diameter of fiber, etc.*

**REPLY R1.3:**

Concerning the fiber diameter and numerical aperture this raises the more general question whether there is a dependence on the number of modes supported by the fiber, which is proportional to the square of the fiber radius multiplied by NA. In principle fibers of higher numerical aperture (or a bigger core) can also be controlled in this manner, at the expense of a longer measurement time. In our experiment, to acquire a single speckle pattern during the calibration step, the total exposure time of the camera was 72 ms and the SLM was given 150 ms to refresh between each step to ensure no cross-talk. Therefore the total time required to sample 625 modes with 4 phase steps is approximately 9 minutes. The computational step for 900 phase masks was completed in approximately 5 minutes. As the number of modes increases the time for both steps would increase and quickly become unmanageable. In principle, the computational step can be completed much faster by working directly in the Fourier plane of the input facet and taking the inverse Fourier transform of the measured transmission matrix $p_{opt,SP}^{j_{x},j_{y},(x_{out},y_{out})}$ (as opposed to our method based on direct summation of the gratings). However the refresh rate of the SLM and acquisition time of the CCD would still represent a bottleneck. Alternatively, a DMD may be used in place of an SLM as it may be refreshed much faster however. However, also reduces the overall power efficiency of the system considerable (Sergey Turtaev, Ivo T. Leite, Kevin J. Mitchell, Miles J. Padgett, David B. Phillips, and Tomáš Čižmár, Optics Express, 2017). We have better highlighted this constraint in the revised version of the manuscript.

The numerical aperture of the input microscope objective determines the resolution at which $B_{scan}^{j_{x},j_{y}}$ can sample the input facet. Ultimately, provided the objective may excite as many pairs as ($j_{x},j_{y}$) as modes of the fiber it does strongly impact the focusing efficiency (see 1.1). Increasing the NA mismatch between the fiber and input objective also reduces the overall transmission of the system.

Overall, the quality of the output reference beam is clearly the most important factor in terms of the resultant focusing efficiency. The data shown in figure 2 and and figure 3 demonstrate that when the output reference beam is more varied in terms of amplitude profile, it is less interformetically compatible with the sampling beam and thus focusing efficiency is lost.

An additional parameter we can investigate is the effect of fiber length. Figure R1.1 shows the outcome of a measurement on a fiber approximately 60 cm long with the reference exciting different sub-regions of the fiber. The output reference beams $f_{SP}(B_{ref}^{i})$ are significantly more uniform in intensity and consequentially the focussing efficiency maps also appear more uniform. However, traces of the profile of $f_{SP}(B_{ref}^{i})$ are visible in the focussing efficiency maps and thus, even in this case, an edge reference appears to result in a more uniform focussing efficiency. However, ultimately, in a longer fiber, the resultant focussing efficiency is less dependent on internal reference position.

***Figure R1.1*** *– Analysis of calibrations based on internal reference beams through a 60 cm long fiber. a) Four internal reference beams at the input facet* $B_{ref}^{i}$*(scale bar 10*$\mu$*m). B) The transmitted internal reference beams* $f_{FP}(B_{ref}^{i} )$ *imaged on CCD2, the fiber core is shown as a red circle. C) Focussing efficiency across the fiber core for each calibration D) Dependence of focussing efficiency on distance from center of the core. E) Histograms of focussing efficiency (for foci within the fiber core). F) The fall in average focusing efficiency between edge and centre reference based calibrations.*

The following text has been added to the manuscript and a new supporting figure (Figure S1) has been added (and shown here as Figure R1.1):

“*The uniformity of the resultant focussing efficiency map appears to be directly related to the the intensity profile of the reference beam. Going further, we have investigated the impact of fiber length and have performed calibrations akin to that in Figure 2 for a fiber approximately 60 cm long. Results are reported in figure S1. As in the previous dataset, the reference beam was evenly shifted (by approximately 6* $\mu$*m) along the radial direction from the very edge to the centre of the fiber core (left to right in Figure S1). The resultant output speckle* $f_{SP}(B_{ref}^{i})$ *are shown in S1B. Although there is still clearly some preservation, the resultant intensity distributions are much more uniform than that for 4 cm fiber. This is in turn reflected in the focussing efficiency maps which are also more uniform for the central reference beam (comparing the far right panels of Figure 2c and Figure S1C ). For both the 4 cm and 60 cm fibers, an edge reference results in higher average focussing efficiency across the fiber core, however this is significantly more pronounced for the 4 cm fiber. Overall, the average focussing efficiency over the core was slightly higher for the short fiber for all reference beams and the average focussing efficiency was much less sensitive to the choice of reference beam for the longer fiber.*”

**QUESTION R1.4:** *Can the blind spots of the inner reference beam be eliminated completely?*

**REPLY R1.4:**

In the original text the experiment shown in Figure 4 supports the hypothesis that the number of blind spots can be reduced by increasing the number of internal references *n_ref_* (from 10/900 for *n_ref_*=1 to 1/900 for *n_ref_=*6). We have repeated this experiment and the new data showed that number of blind spots for a fiber length of 4 cm is N*_blind_spots_*=8.67$\pm$4.16 for *n_ref_*=1, while for *n_ref_*=6 we obtained average value N*_blind_spots_*=0.33$\pm$0.58 (N*_blind_spots_* is evaluated as mean $\pm$ standard deviation for n=3 trials). Therefore, for our configuration we estimate that the optimal number of internal references would be approximately *n_ref_* =6. The new data are shown in Figure R1.2 for the reviewers’ convenience and reported in an amended Figure 4.

New data concerning the generation of multiple foci simultaneously has also been added in the final panel.

***Figure R1.2*** *(Figure 4 in main text)– Analysis of calibrations based on serialised internal reference beams. A) Characteristic example of a focussed spot on the fiber surface (top) and a blind spot (bottom) B) Focussing efficiency for four serialised internal reference based calibrations based on single reference (top left), two references (top right), three references (bottom left) and six references (bottom right) C) Histograms of the focussing efficiency for the four measurements, the inset shows the highlighted region which were classified as blind spots. D) The average number of blind spots in each calibration (left y-axis) and the blind spot coverage ratio (no of blind spots/no of focussed spots) (right y-axis), based on 3 experiments. E) Multi-spot focussing through a MMF based on single (top) and multiple (bottom) internal reference calibrations.*

And text:

*“The average number of blind spots falls from 8.67±4.16 with a single reference calibration to 0.33±0.58 for the 6 reference beam based calibration (based on n=3 experiments).”*

**QUESTION R1.5:** *In Figures 2 and 3, the scale bar is not indicated. In Figure 3(e), the number “35” is blocked.*

**REPLY R1.5:**

We have amended the figure caption to include the length of scale bars and circles identifying fiber core/NA shown throughout and the error in 3E is fixed.

**QUESTION R1.6:** *In Figure 3, what do the theta_u and theta_v refer to?*

**REPLY R1.6:**

We thank the reviewer for noting the error in notation here and have switched $\theta$*_u_* and $\theta$*_v_* to *u_out_, v_out_*

REVIEWER 2

**COMMENT:**

*In the manuscript tilted “Determining the optimal reference beam to measure the transmission matrix of a multimode optical fiber”, the authors present the merits of using internal reference method to improve focusing through a multimode fiber. The internal reference method is a great idea in my opinion, but the work presented by the author needs to ‘dig a bit deeper’.*

*The best way to generate a single focus using wavefront shaping method is the step sequential method introduced at the dawn of the technique. The transmission matrix method is the most accurate but it requires determining both amplitude and phase of the transmitted signal. The most accurate method to obtained the TM uses some sort of external/stable reference beam (holographic method) or uses phase retrieval techniques (a bit more computationally intensive). All the above methods can generate/transfer images through multimode fibers.*

*Here the authors only demonstrate their work for a single focus. I believe Collard et al. will have to put their work more in context to justify this conclusion “Overall, our study demonstrates the utility of internal reference-based systems, which is inherently reconfigurable, we envisage this will be crucial in allowing the technology to reach its full potential for hyper-spectral imaging at the tip of a hair thin optical fiber.”*

*See below for further questions and concerns:*

**REPLY:**

We thank the reviewer for his thorough reading of the manuscript and positive overall assessment of the work. To better demonstrate the utility of our approach several new experiments have been performed and the discussion has also been revised to put the results more in context with the conclusions. We have also improved our comparison between other methods (external reference, direct phase measurement and machine learning).

New experiments have been performed on longer fibers, multiple foci have been generated simultaneously and the phase profile of internal reference has been measured. These experiments (detailed below) support the hypothesis that our method can be translated to longer fibers and also to transmit more complex patterns, all with the same “optical setup” based on a common optical path.

We hope that the new experimental data and the modified narrative has improved the quality of our study. Please find below a detailed point by point response.

**QUESTION R2.1:** *The authors can do a little bit more of digging in the literature. There is quite a comprehensive body of work for wavefront shaping in multimode fiber both with external reference, internal reference, and reference-less (phase retrieval). How is this method compare with the other well established endoscopic imaging method?*

**REPLY R2.1:**

We thank the reviewer for bringing this to our attention. The introduction has been edited to provide a more accurate representation of the state of the art, in particular by citing works focusing on reference-less techniques.

*“Therefore, as the field develops and more complex multispectral imaging modalities, simplified transmission matrix measurements methods are of keen interest. In this respect, “reference free”, phase retrieval techniques [25,26] and machine learning algorithms [27,28] have both emerged as elegant alternatives, as they require no kind of interferomic measurement however they generally require larger datasets/more complex computational techniques and are generally less accurate than interferomic measurements”*

*25.	M. N’Gom, T. B. Norris, E. Michielssen, and R. R. Nadakuditi, "Mode control in a multimode fiber through acquiring its transmission matrix from a reference-less optical system," Opt. Lett. 43, 419–422 (2018).*

*26.	T. Zhao, L. Deng, W. Wang, D. S. Elson, and L. Su, "Bayes theorem-based binary algorithm for fast reference-less calibration of a multimode fiber," Opt. Express 26, 20368–20378 (2018).*

*27.	B. Rahmani, D. Loterie, G. Konstantinou, D. Psaltis, and C. Moser, "Multimode optical fiber transmission with a deep learning network," Light Sci. Appl. 7, 69 (2018).*

*28.	B. Rahmani, D. Loterie, E. Kakkava, N. Borhani, U. Teğin, D. Psaltis, and C. Moser, "Actor neural networks for the robust control of partially measured nonlinear systems showcased for image propagation through diffuse media," Nat. Mach. Intell. 2, 403–410 (2020).*

**QUESTION R2.2:** *The authors should revised their references. For example Reference [24] is not a reference less method. They have an external reference going through a second fiber. I do not believe they claim AI. The references for [25] and [26] aren’t accurate either. I believe the numbers have been shifted down. Please revise.*

**REPLY R2.2:** We are grateful to the reviewer for identifying this indexing error and have amended it.

**QUESTION R2.3:** *The length of the fiber (4 cm) used in this experiment is of concern. As far as I know, a lot of the single fiber endoscopic method in the literature have much longer fibers, some with much larger core (more modes). How will this method presented here, work when the fiber is 10 or 20 times longer? This isn’t obvious to me; in a longer fiber the light are even more scrambled. Further, bends, temperature, vibrations, etc. will also affect both the signal and reference. How is this accounted for in this study?*

**ANSWER R2.3:** We have also investigated the possibility of working with longer optical fibers. Figure R1.1 shows the outcome of a measurement on a fiber approximately 60 cm long with the reference exciting different sub-regions of the fiber. The output reference beams $f_{SP}(B_{ref}^{i})$ are significantly more uniform in intensity and consequentially the focussing efficiency maps also appear more uniform. However, traces of the profile of $f_{SP}(B_{ref}^{i})$ are visible in the focussing efficiency maps and thus, even in this case, an edge reference appears to result in a more uniform focussing efficiency. However, ultimately, in a longer fiber, the resultant focussing efficiency is less dependent on internal reference position.

We would also like to highlight that endoscopic imaging applications in the mouse brain are usually performed with short fiber stubs [S. Turtaev, I. T. Leite, T. Altwegg-Boussac, J. M. P. Pakan, N. L. Rochefort, and T. Čižmár, "High-fidelity multimode fibre-based endoscopy for deep brain in vivo imaging," Light Sci. Appl. 7, 92 (2018).] [ S. A. Vasquez-Lopez, R. Turcotte, V. Koren, M. Plöschner, Z. Padamsey, M. J. Booth, T. Čižmár, and N. J. Emptage, "Subcellular spatial resolution achieved for deep-brain imaging in vivo using a minimally invasive multimode fiber," Light Sci. Appl. 7, 110 (2018)].

***Figure R2.1*** *– Analysis of calibrations based on internal reference beams through a 60 cm long fiber. a) Four internal reference beams at the input facet* $B_{ref}^{i}$*(scale bar 10*$\mu$*m). B) The transmitted internal reference beams* $f_{FP}(B_{ref}^{i} )$ *imaged on CCD2, the fiber core is shown as a red circle. C) Focussing efficiency across the fiber core for each calibration D) Dependence of focussing efficiency on distance from center of the core. E) Histograms of focussing efficiency (for foci within the fiber core). F) The fall in average focusing efficiency between edge and centre reference based calibrations.*

The following text has been added to the manuscript and a new supporting figure (Figure S1 has been added (and shown here as Figure R1.1).

“*The uniformity of the resultant focussing efficiency map appears to be directly related to the the intensity profile of the reference beam. Going further, we have investigated the impact of fiber length and have performed calibrations akin to that in Figure 2 for a fiber approximately 60 cm long. Results are reported in figure S1. As in the previous dataset, the reference beam was evenly shifted (by approximately 6* $\mu$*m) along the radial direction from the very edge to the centre of the fiber core (left to right in Figure S1). The resultant output speckle* $f_{SP}(B_{ref}^{i})$ *are shown in S1B. Although there is still clearly some preservation, the resultant intensity distributions are much more uniform than that for 4 cm fiber. This is in turn reflected in the focussing efficiency maps which are also more uniform for the central reference beam (comparing the far right panels of Figure 2c and Figure S1C ). For both the 4 cm and 60 cm fibers, an edge reference results in higher average focussing efficiency across the fiber core, however this is significantly more pronounced for the 4 cm fiber. Overall, the average focussing efficiency over the core was slightly higher for the short fiber for all reference beams and the average focussing efficiency was much less sensitive to the choice of reference beam for the longer fiber.*”

**QUESTION R2.4:** *Further, the most accurate holographic techniques use a spatially filtered external reference beam: the phase and intensity of the beam well controlled and known. The idea of an internal reference is indeed fascinating, but the internal reference will also suffer the same fate as the signal beam. Can the author discuss how is the amplitude and phase of the internal reference determined? In equation (3) in the manuscript, the intensity of the reference beam is measured accurately, but how is the phase determined?*

**ANSWER R2.4:** The reviewer is correct to identify that the interferometric compatibility of B_ref_ and B_Scan_ is indeed impacted by the amplitude and that the phase of the output reference speckle is also scrambled by the fiber. Therefore, the output foci are no longer phase matched (as they would be in an external reference based calibration as the external reference has constant phase).

Using an external reference path and the phase unwrapping technique [I. N. Papadopoulos, S. Farahi, C. Moser, and D. Psaltis, "Focusing and scanning light through a multimode optical fiber using digital phase conjugation," Opt. Express 20, 10583–10590 (2012).] we have measured the phase profile of the internal reference beam (and shown here as Figure R2.2 and figure S2 in the main text). The focusing efficiency map does not appear to be strongly correlated to features in the phase profile. Each focused spot represented in the FE map (A) is now aligned to the phase of the corresponding point in the phase profile of the reference. Therefore unlike the external reference measurement (where all the focused spots would be phase aligned), the foci on the surface vary in phase profile.

***Figure R2.2*** *– A) The resultant focussing efficiency map from an internal reference (edge) based calibration. B) The phase profile of the internal reference measured by a phase unwrapping of the interferogram between the internal and external reference beams.*

*“The phase profile of the internal reference beam was measured using an external reference and the phase unwrapping technique described in  [3]. The phase profile is shown in figure S2A and the resultant focussing efficiency map is shown in S2B. No correlations between the phase pattern of the internal reference and the focusing efficiency map can be identified, however it is noteworthy that the output foci are no longer phase aligned (as they would be for a uniform phase profile external reference).”*

**QUESTION R2.5** *How is the work presented in this manuscript improve from what has been done in reference [26]? What is novel? Different?*

**ANSWER R2.5:** We thank the reviewer for allowing us to better explain the differences of our work with respect to ref [26] (P. Jákl, M. Šiler, J. Ježek, Á. Cifuentes, J. Trägårdh, P. Zemánek, and T. Čižmár, "Endoscopic Imaging Using a Multimode Optical Fibre Calibrated with Multiple Internal References," Photonics 9, (2022)), which is focussed on establishing the merits of using multiple reference beams in sequence. While we have also tested the serialised reference beam, our work is has a specific focus on investigating the optimal internal reference beam both in terms of amplitude profile and positional on the input facet. Overall, our conclusions are in agreement with this work, but are obtained from a different experimental perspective: determining the overall optimal reference. We feel the new experimental data added during the peer review provide a more clear differentiation of the revised manuscript with respect to ref 26.

**QUESTION R2.6***. There is a discussion on a single focus. Wavefront shaping have significantly moved on to multiple foci and image transfer. How will the changes in a multiple internal reference affect a multiple foci problem? An image?*

**ANSWER R2.6:** New data has been added to the figure 4 in the main text showing how multiple foci can be generated simultaneously based on a multiple internal reference calibration and a single internal reference calibration and reproduced for the reviewer’s convenience here (Figure R2.3). In this experiment, the focusing efficiency is effectively shared between the two foci independently of the number of references used. This is because when the phase masks are merged, the amplitude information is effectively discarded (as the SLM is phase only).

***Figure R2.3*** *(Figure 4 in main text)– Analysis of calibrations based on serialised internal reference beams. A) Characteristic example of a focussed spot on the fiber surface (top) and a blind spot (bottom) B) Focussing efficiency for four serialised internal reference based calibrations based on single reference (top left), two references (top right), three references (bottom left) and six references (bottom right) C) Histograms of the focussing efficiency for the four measurements, the inset shows the highlighted region which were classified as blind spots. D) The average number of blind spots in each calibration (left y-axis) and the blind spot coverage ratio (no of blind spots/no of focussed spots) (right y-axis), based on 3 experiments. E) Multi-spot focussing through a MMF based on single (top) and multiple (bottom) internal reference calibrations.*

The following text has been added to the manuscript and a new panel added to figure 4:

*Figure 4E shows how two foci may be generated simultaneously at two output points with both a single and multiple reference calibration following the formula:*

$$\Phi_{SP}^{\left( x_{1},y_{1} \right),\left( x_{2},y_{2} \right)}=arg(\exp\left( i\Phi_{SP}^{(x_{1},y_{1})} \right)+exp(i\Phi_{SP}^{(x_{2},y_{2})}))$$

*With the focusing efficiency shared between them. As this computation discards the amplitude information, the intensity distribution is not impacted by the number of reference beams used to calibrate.*

**QUESTION R2.7***. I hope the authors will find my reviews helpful and hopefully improve the manuscript*

We thank Dr N'Gom for his thorough reading of the manuscript and constructive peer review.

REVIEWER 3

**COMMENT:**

*In this manuscript, the authors investigated the conditions that affect the optimization of reference beams for multi-mode optical fiber’s transmission matrix measurements based on the common-path interferometric method. The authors defined the focusing efficiency and used this as a metric to experimentally investigated the optimal parameters for determining the internal reference beam. Related experimental data can support their claims. This work helps establish the metrics and method to optimize the reference beam as well as investigate the optimal experimental scheme to this end. Since wavefront-shaping-based optical fiber transmission matrix calibrations play a central role in fiber imaging, this work can benefit related fiber-based biomedical imaging research to a large extent. Overall, I would like to recommend the publication of this work. However, The writing of this manuscript has some minor issues. The authors need to address the following minor issues in their revised manuscript.*

**REPLY:**

We thank the reviewer for the positive opinion on the work and the constructive comments. Please find below our answers to the different comments and suggestions.

**QUESTION R3.1:***. In the authors’ definition of focusing efficiency, it is not quite clear how the authors define “dark currents”? The physics meaning of the “dark currents” should be clarified.*

**ANSWER R3.1:** We thank the reviewer for identifying this. We have amended the text and now refer to this as “background electrical noise”

*“The quality of the calibration was therefore analysed by estimating the focussing efficiency (FE) across the output facet, defined as* $FE=\frac{I_{foci}-\varepsilon_{foci}}{I_{total}-\varepsilon_{total}}$ *where* $I_{foci}$ *is the intensity within the SLM-generated foci,* $I_{total}$ *is the total intensity transmitted by the fiber and* $\varepsilon_{foci}$ *and* $\varepsilon_{total}$ *are the total readout electrical noise of the CMOS camera in the foci and in the entire facet, respectively. In each calibration, a 30 by 30 array of foci was targeted at the output facet.”*

**QUESTION R3.2** *In Figure 1, there are supposed to be four focal planes. However, in the experimental system drawing, only three focal planes are illustrated.*

**ANSWER R3.2:** We thank the reviewer for identifying this. It has been clarified both in the figure and caption that *(u_in_, v_in_), (x_in_, y_in_)*, *(x_out_, y_out_)*, *(u_out_, v_out_)* correspond to the screen of the SLM, proximal fiber facet, distal fiber facet, distal Fourier plane. We added the following text:

*“The four focal planes (u_in_, v_in_), (x_in_, y_in_), (x_out_, y_out_), (u_out_, v_out_) correspond to the screen of the SLM, proximal fiber facet, distal fiber surface plane, distal Fourier plane and are indicated by the purple, blue, green and brown dashed lines respectively”*

**QUESTION R3.3** *All the figures in this manuscript use upper-case letters. However, when the authors cited the figures in their main text, they used lowercase letters, which should be corrected.*

**ANSWER R3.3:** This has been amended and the figure index’s are now upper case.

**QUESTION R3.4** *The authors should add the length of the scale bar in Figure 2 and Figure 3.*

**ANSWER R3.4:** Thanks, the length of scale bars have been added to the captions.

**QUESTION R3.5** *Figure 2 d is confusing. Curves with different colors indicate focusing efficiency correlated with different spatial locations. Then, what is the meaning of the horizontal axis?*

**ANSWER R3.5:** We thank the reviewer for identifying this. It should be noted that the x axis “radial distance” refers to radial distance of the foci in the ***output facet*** i.e. $\sqrt{{x_{out}}^{2}+{y_{out}}^{2}}$. The color scale refers to the reference position in the ***input facet*** i.e.$\sqrt{{x_{in}}^{2}+{y_{in}}^{2}}$. We recognize this as potential source of confusion and have relabeled the x axis as $\sqrt{{x_{out}}^{2}+{y_{out}}^{2}}$

REVIEWER 4

**COMMENT:**

*In the article the authors investigate to achieve desired amplitude distributions at the tip of a multimode*

*fiber (MMF) by using pre-shaped light. The model is extremely simple: they use two sawtooth gratings with some phase shift between together. It allows to control a little bit the intensity and the output of short 4-cm long MMF. The work has a lot of drawbacks that should be fixed before publication:*

**REPLY:**

We thank the reviewer for his/her review of our work and the constructive comments. We hope that with the new data and revised narrative, the reviewer will now consider the work appropriate for publication in PLOS one.

**QUESTION R4.1:** *The title is confusing. In fact the authors did not present a measured transmission matrix as is has been done in other works like [1-3].*

**REPLY R4.1:** We recognize that our method does not directly measure the transmission matrix of the MMF and have amended the title and text accordingly.

New title :

***Optimizing the internal phase reference to shape the output of a multimode optical fiber***

**QUESTION R4.2:** *In the abstract the authors refer to optically turbid waveguide, but a standard MMF that is absolutely transparent for light was used in the experiments. What did it really mean?*

**REPLY R4.2:** By turbidity we are referring to the scrambling of the structured input light into an effectively random speckle pattern after transmission, which makes the fiber a turbid medium [Cizmar. T., Dholakia. K., Optics Express 2011]. To avoid confusion, we have clarified this in the abstract:

“*Such techniques rely on measuring the transmission matrix of the optically turbid waveguide which scrambles the coherent input light into an effectively random speckle pattern. Typically, this is done by measuring the interferogram between the output speckle and a reference beam*”

**QUESTION R4.3:** *SP and FP planes have not denoted in the Fig.1 but mentioned in the text.*

**ANSWER R4.3:** Thanks, we have fixed this and added into the text:

“*The four focal planes (u_in_, v_in_), (x_in_, y_in_), (x_out_, y_out_), (u_out_, v_out_) correspond to the screen of the SLM, proximal fiber facet, distal fiber surface plane, distal Fourier plane and are indicated by the purple, blue, green and brown dashed lines respectively”*

**QUESTION R4.4:***Why the authors use such simply expression and do not mention other more improved methods like [4] for scanning?*

**ANSWER R4.4:** We thank the reviewer for bringing this to our attention, we recognize that by directly modulating in the Fourier plane, the speed at which the phase masks $\Phi_{SP}^{(x_{out},y_{out},j_{ref})}\left( u_{in},v_{in} \right)$ and $\Phi_{FP}^{(x_{out},y_{out},j_{ref})}\left( u_{in},v_{in} \right)$ can be computed can potentially be increased with such a method. We have added the following text in the experimental method acknowledging this point.

*“This computation was then completed on the GPU for all the output foci in both SP and FP. This computational step took approximately 5 minutes for all 900 phase masks. Thus the total time for a single internal reference measurement was approximately 14 minutes. In principle it can completed faster by taking the inverse Fourier transform of the measured transmission matrix* $p_{opt,SP}^{j_{x},j_{y},(x_{out},y_{out})}$ *or* $p_{opt,FP}^{j_{x},j_{y},(x_{out},y_{out})}$*.“*

**QUESTION R4.5** *5. The phase shift 'p' is unclear and there is no equation how exactly it affects to the sawtooth gratings.*

**ANSWER R4.5:** We thank the reviewer for identifying this typographical error in the equation. We have amended accordingly:

*“Prior to the calibration, sawtooth gratings* $\phi_{scan}^{j_{x},j_{y},p}(u_{in},v_{in})=mod ({au}_{in}+{bv}_{in}+p,2\pi)$ *and*$\phi_{ref}\left( u_{in},v_{in} \right)=mod \left( {(cu}_{in}+{dv}_{in} \right),2\pi)$ *were defined, where a and b set the periodicity of the grating along* $x_{in},$ *and* $y_{in}$ *so the beam* $B_{scan}^{j_{x},j_{y},p}$ *raster scans a 25 × 25 grid overlaying the input facet of the MMF indexed by (*$j_{x},j_{y}$*)”.*

**QUESTION R4.6***. 6. Which array of points was used, 25x25 or 30x30? Both of them can be found in the text.*

**ANSWER R4.6:** The 25 by 25 array of points refers to indexing at the input ($j_{x},j_{y}$) whereas the 30 by 30 array is output points (either in the surface plane or Fourier plane ($x_{out},y_{out}$) ,($u_{out},v_{out}$)). This has been clarified in the text:

“*The first part of the calibration consisted of measuring the transmission of* $B_{interfere}^{j_{x},j_{y},p}(u_{in},v_{in})$ *through the MMF on the Surface Plane (SP) and Fourier Plane (FP) of the output facet, hereafter referred to as* $f_{SP}(B_{interfere}^{j_{x},j_{y},p}$*) and* $f_{FP}(B_{interfere}^{j_{x},j_{y},p}$*), for each value* $j_{x},j_{y},p$ *at a 30 × 30 set of points in both output planes (*$x_{out},y_{out}$*) ,(*$u_{out},v_{out}$*) which were measured on CCD2 and CCD 3 respectively.*”

**QUESTION R4.7***. 7. The used MMF was only 4-cm long. Are there any applications where it would be in demand? For me such length is impractical.*

**ANSWER R4.7** Endoscopic imaging applications in the mouse brain are usually performed with short fiber stubs [*S. Turtaev, I. T. Leite, T. Altwegg-Boussac, J. M. P. Pakan, N. L. Rochefort, and T. Čižmár, "High-fidelity multimode fibre-based endoscopy for deep brain in vivo imaging," Light Sci. Appl. 7, 92 (2018).] [ S. A. Vasquez-Lopez, R. Turcotte, V. Koren, M. Plöschner, Z. Padamsey, M. J. Booth, T. Čižmár, and N. J. Emptage, "Subcellular spatial resolution achieved for deep-brain imaging in vivo using a minimally invasive multimode fiber," Light Sci. Appl. 7, 110 (2018)*].

In the revised version of the manuscript we have also investigated the possibility of working with longer optical fibers. The figure R1.1 shows the outcome of a measurement of a fiber approximately 60 cm long with the reference exciting a different sub-region of the fiber. The output reference beams$f_{SP}(B_{ref}^{i})$, are significantly more uniform in intensity and consequentially the focussing efficiency maps also appear more uniform. However, traces of the profile of $f_{SP}(B_{ref}^{i})$ are visible in the focussing efficiency maps and thus, even in this case, an edge reference appears to result in a more uniform focussing efficiency. However, ultimately, in a longer fiber, the resultant focussing efficiency is less dependent on internal reference position.

The following text has been added to the manuscript and a new supporting figure (Figure S1) has been added (and shown here as Figure R1.1).

“*The uniformity of the resultant focussing efficiency map appears to be directly related to the the intensity profile of the reference beam. Going further, we have investigated the impact of fiber length and have performed calibrations akin to that in figure 2 for a fiber approximately 60 cm long in figure S1. As in the previous dataset, the reference were evenly shifted (by approximately 6* $\mu$*m) along the radial direction from the very edge to the centre of the fiber core (left to right in the figure S1). The resultant output speckle* $f_{SP}(B_{ref}^{i})$*, are shown in S1B, although there is still clearly some preservation, the resultant intensity distributions are much more uniform than that for 4 cm fiber. This is in turn reflected in the focussing efficiency maps which are also more uniform for the central reference beam (comparing the far right panels of Figure 2c and Figure S1C ). For both the 4 cm and 60 cm fibers, an edge reference results in higher average focussing efficiency across the fiber core however this is significantly more pronounced for the 4 cm fiber. Overall, the average focussing efficiency over the core was slightly higher for the short fiber for all reference beams and the average focussing efficiency was much less sensitive to the choice of reference beam for the longer fiber.*”

*Figure R.1 – Analysis of calibrations based on internal reference beams through a 60 cm long fiber. a) Four internal reference beams at the input facet* $B_{ref}^{i}$*(scale bar 10*$\mu$*m). B) The transmitted internal reference beams* $f_{FP}(B_{ref}^{i} )$ *imaged on CCD2, the fiber core is shown as a red circle. C) Focussing efficiency across the fiber core for each calibration D) Dependence of focussing efficiency on distance from center of the core. E) Histograms of focussing efficiency (for foci within the fiber core). F) The fall in average focusing efficiency between edge and centre reference based calibrations.*

**QUESTION R4.8***. The first paragraph on page 9 describes the figure from supplementary that is really strange... It would be better to modify Fig1 to show external reverence beam and add one more figure with the characteristics of the focal point or prepare self-consistent supplementary.??*

**ANSWER R4.8:** We thank the reviewer for this comment.,we have modified figure 1 to show the external reference path.
