# Supplementary material for: Optimizing the internal phase reference to shape the output of a multimode optical fiber
Source: PLoS One. 2023 Sep 8;18(9):e0290300. doi: 10.1371/journal.pone.0290300 (PMC10490902; doi:10.1371/journal.pone.0290300)
Supplement: Attachment — Submitted filename: ResponseLetterTWO.docx [file pone.0290300.s004.docx]

REVIEWER 5

**COMMENT:**

*In this manuscript, the authors perform assessments for both surface and Fourier plane calibrations. In the surface calibration, they focus the light on the distal facet, and in the Fourier plane calibration, they collimate the light into a low-divergence beam emerging from the distal facet. To optimize the system's relative focusing efficiency, they employ both single and serialized internal reference beams. This work may deserve publication in my opinion.*

*However, several revisions, including some major ones, still need to be addressed.*

**REPLY:**

We thank the reviewer for the positive opinion on the work and thorough reading of the work, which have helped us in improving the overall readability and quality of the manuscript. Our detailed answers are reported below.

**QUESTION R1.1:** *The authors should carefully check their notations as there are several unacceptable notation errors in the manuscript. Some are listed below:*

*1a) On page 26, it should be "(u_in, v_in)" instead of "(u_in, y_in)."*

*1b) In Figure 2D, the x-axis should be labeled as "\sqrt{x_out^2, y_out^2)}" instead of "\sqrt{x_out^2, v_out^2)."*

*1c) On page 34, the correct notation for the calibration in the Fourier plane is "(u_out, v_out)" rather than "(u_out_v_out).".*

**REPLY R1.1:**

We thank the reviewer for identifying these error which have been resolved and highlighted

**QUESTION R1.2:** *Figure S3 has been incorrectly labeled as Figure S1 in the Supporting Information. To rectify this error, the authors must ensure that the correct label, "Figure S3," is used for the respective figure in the Supporting Information section. Moreover, it is essential to update the caption in Figure S3 to accurately correspond to the content of Figure S3*

**REPLY R1.2:**

We thank the reviewer for identifying this error and have amended it and changed the caption of figure S3:

**QUESTION R1.3:** *The meaning of "S1B" on page 31 and "S2B" on page 32 is unclear. Should they be Figure S1B and Figure S2B?*

**REPLY R1.3:**

This has been amended and all labels are now formatted “Figure S1A”

**QUESTION R1.4:** *Following QUESTION R3.2, the focal planes for (u_in, v_in) and (u_out, v_out) in Figure 1A are challenging to discern. To address this issue, the authors should consider modifying the colors used for these focal planes to enhance visibility and clarity.*

**REPLY R1.4:**

This has been amended and these focal planes are now coloured orange and pink (respectively).

**QUESTION R1.5:** *Following QUESTION R3.3, the issue has not been fully addressed yet. For example, Figure 2b on page 29 and Figure 2c on page 32 still require corrections. Additionally, there are inconsistencies in the citation format within the main text, where certain figure references are bolded while others are not. Moreover, the authors sometimes use "Figure S1" and other times "figure S1."*

**REPLY R1.5:**

This has been amended and all labels are now formatted “**Figure S1A**”

**QUESTION R1.6:** *) The authors should include a legend to distinguish between the two different colors in the histograms for both Figure 2E and Figure 3E.*

**REPLY R1.6:**

A legend has been added to both panels indicating the reference beam used to calibrate: Central reference- CR Upper intermediate reference – UIR Lower intermediate reference – LIR Edge reference -ER

**QUESTION R1.7:** *In addition to mentioning the information in the caption, the authors should also label the number of references in Figure 4B.*

**REPLY R1.7:**

Labels have been added to each panel

**QUESTION R1.8:** *The "um" in the x-axis of Figure S1D should be corrected to "μm."*

**REPLY R1.8:**

This has been corrected.

**QUESTION R1.9:** *The caption of Figure 2 requires revision to include the scale bar information for Figure 2A, which is currently missing. It's important to note that the mentioned 10 m scale bar is not present in Figure 2C, despite being referenced in the caption.*

**REPLY R1.9:**

This has been amended

**QUESTION R1.10:** *The colorbar label in Figure 3C, Figure S2A, Figure S2B, and Figure S3B should be horizontally flipped to make them consistently.correct notation for the calibration in the Fourier plane is "(u_out, v_out)" rather than "(u_out_v_out).".*

**REPLY R1.10:**

This has been corrected

**QUESTION R1.11:** *The authors should be mindful of their use of abbreviations to prevent redundancy. For example, on page 28, they introduce the abbreviation "SP" for "surface plane." However, on page 29, they opt to abbreviate "surface plane" once more instead of using the established abbreviation "SP."*

**REPLY R1.11:**

This error has been amended.

**QUESTION R1.12:** *There are punctuation errors that need to be addressed. In two instances (pages 26 and 28), the authors used ".." to end sentences, but it should be corrected to use ".", instead. Additionally, on page 31, the authors used ",." which should also be fixed to "." for proper punctuation.*

**REPLY R1.12:**

These have been amended.
